# Does the Arc of Science Bend Towards Impact? Four Decades of Empirical Research Published in JADD Since the DSM-III

**DOI:** 10.1007/s10803-021-05052-2

**Published:** 2021-05-11

**Authors:** Peter Doehring

**Affiliations:** ASD Roadmap, 5 Nine Gates Road, Chadds Ford, PA 19317 USA

**Keywords:** Autism spectrum disorder, Review, Publication trends, Basic research, Applied research, Intervention research, Community programs

## Abstract

The present study explored the shift from understanding to intervention to population impact in the empirical research published in this journal at five points of time over 40 years since the release of DSM-III. Two-thirds of the more than 600 original studies identified involved basic research, a pattern that is consistent with previous analyses of research funding allocations and that did not change over time. One of every eight studies involved intervention research, which occurred in community-based programs only about one-quarter of the time. These gaps in intervention research and community impact did not improve over time. The findings underscore the need to broaden the training and experience of researchers, and to re-consider priorities for research funding and publication.

The pre-eminent role that science plays in advancing the goals of our society is without dispute. Basic research studies rely on rigorous observation to describe and explore the potential significance of new phenomena, and then test specific hypotheses through systematic experimentation. Over time, applied research studies begin to explore how to translate this new understanding into more practical uses that improve our lives in meaningful and measurable ways. If these efforts are successful, we might eventually document the positive impacts on society as a whole. Yet for all of our investments in science, the scientific process itself is rarely the object of study. What could we learn if we use the tools of science to understand how research in a specific field grows and changes over time? Does the arc of research bend over the decades from understanding to implementation and eventually to societal impact?

One way that we have tried to capture elements of this evolution for conditions like Autism is through revisions to systems for classifying conditions, like the Diagnostic and Statistical Manual of Mental Disorders (DSM), now in its 5th edition. Consider changes to the classification of autism over this period. Autism first emerged as an occasional characteristic of childhood schizophrenia in the first edition published in (1952), and by the second edition (1968) was recognized as a more distinct subtype of schizophrenia that is sometimes accompanied by mental retardation. Autism only emerged as a distinct developmental disorder in the third edition (1980). The fourth edition (1994) expanded the number of subtypes, only to have these collapsed into a single spectrum disorder in the fifth edition (2013). Each of these changes was spurred by new research and understanding into the characteristics, course, and possible causes of ASD. For example, changes introduced by the DSM-III reflected the growing consensus that Autism was more likely to result from differences found in genetics and neurology rather than the parental relationship (Bettleheim, 1967), while subsequent changes sought to align subtypes with differences discovered through research. With each new insight into the characteristics, course, and possible causes of ASD came new hope for better interventions and ultimately better outcomes. Have these distinctions proved to be meaningfully different?

This special edition of the journal, reflecting back over the four decades since Autism was recognized as a distinct developmental disorder in the DSM-III, provides an opportunity to shed some light on the scientific process itself, at least with respect to autism. The goal of this study was to explore whether patterns in the empirical research published in this journal, at five points of time, also reflect a gradual shift from understanding to intervention to population impact. Could this offer a roadmap for research, from the first basic research studies beginning to outline core features to applied research studies that demonstrate the effectiveness of intervention for the individual and the impact on the population at large?

There are several reasons why a research roadmap might be important to capturing the progress that scientists seek. For example, a research roadmap recognizes that scientific understanding typically evolves over time through the cumulative impact of many individual research studies and many inter-related lines of inquiry. This appears especially likely with respect to research that seeks to translate better understanding into better outcomes at the population level through widespread improvements in community-based practice. No single study has revealed causal features, demonstrated an effective intervention, and then documented how such an intervention could be successfully implemented across an entire population.

Reviews that broadly map progress in specific areas of research can be found throughout the pages of this journal across the decades, but few reviewers have sought to systematically characterize an entire body of research along this spectrum of impact. Such reviews tracking the progress of research from basic understanding to implementation and impact in a specific topic might prove revealing. Consider a topic like ASD screening, which explicitly aims to achieve population-level improvements in an outcome that is universally accepted as critical; early identification and intervention. A preliminary application of a roadmap to research surrounding the Checklist for Autism in Toddlers (CHAT) and its variants captured the progression from basic research through stages of applied research scale. Nonetheless, the evidence that these instruments actually contributed to earlier and more accurate diagnosis across an entire region remains unclear.

A research roadmap might also capture progress towards implementation and impact not just in specific areas (like ASD screening) but across an entire area of study (like autism). The discovery of any new feature of autism – whether a new genetic variant, a new neurological structure or function, or a new behavioral characteristic – might spawn multiple research studies to understand the relevance of that feature to outcomes and interventions. It is possible – indeed, even likely—that the number of basic research studies seeking to first characterize this new feature will far outweigh the number of applied research studies seeking to develop interventions, especially in the early stages of discovery, and certainly if relevant interventions prove elusive. A shift towards applied research might begin, however, as research on more promising features yields possible options for assessment and treatment. Such a shift might occur over a period of decades across a broad range of features, to the extent that researchers make progress towards evaluating the potential significance of more and more features of autism. To date, there is little evidence that such a shift has occurred (Bailey, 2009; Zwicker & Emery, 2014).

One group that has broadly characterized the continuum of research from basic understanding to population impact is the Inter-Agency Autism Coordinating Committee (IACC). IACC monitors the allocation of research funding as part of their larger mandate to develop a national strategy for ASD research coordinated across major public and private agencies (Interagency Autism Coordinating Committee, 2020a). Their analyses assign funded research projects to one of seven categories linked to its strategic plan: (a) Screening/Diagnosis, (b) Biology, (c) Risk Factors, (d) Treatments/Interventions, (e) Services, (f) Lifespan Issues, and (g) Infrastructure and Surveillance (Office of Autism Research Coordination, 2019). While these seven categories and related subcategories might be loosely described as a type of roadmap, they have never been analyzed as such. To date, IACC’s reports have been limited to summaries of expenditures (Office of Autism Research Coordination, 2019), annual reports highlighting specific research findings (Interagency Autism Coordinating Committee, 2020b), summaries of the number of publications and collaborations that have resulted (Office of Autism Research Coordination, 2012), and reports to Congress (U.S. Department of Health and Human Services, 2019). There are no publications that have sought to systematically evaluate the impact with respect to the number of publications within categories of research along a continuum, let alone document the improvements in practice, or the impact on individuals living with ASD.

Other researchers have adopted a similar strategy, focusing on analyses of the allocation of research funding. Zwicker and Emery’s (2014) model for evaluating the economic impact of ASD research regrouped the IACC categories of Biology and Risk Factors into a Basic Research Category, and the remaining categories into a Clinical and Translational Research Category. In this analysis, 42% of $408 million spent on research in 2010 would be classified as Basic Research. In a report commissioned by the UK charity Research Autism, Pellicano et al. (2013) applied a similar framework to the patterns of research funding in the UK and US, while also exploring priorities voiced by the autism community in the UK. Their analyses of autism research funding in the UK between 2007 and 2011 revealed a greater emphasis on basic research. The emphasis on basic research was more apparent in publication trends, increasing over time between 2001 and 2011. The majority of stakeholders in the autism community surveyed were dissatisfied, however, with the relative emphasis on basic research.

We have also undertaken analyses of IACC research sub-categories and specific grants to capture the relative focus on different types of research more precisely. We re-analyzed research allocations in 2013 by IACC funding subcategories (Office of Autism Research Coordination, 2017) to focus on Research on Practice and Policy (Doehring, 2019b), which included Assessment, Intervention, Implementation (of community-based services), Prevalence/Surveillance, and Population Outcomes. In contrast, Basic Research included all other research focused on the causes, characteristics, associated features, and developmental trajectory of ASD: the broad categories of Biology and Risk Factors; all subcategories under Infrastructure and Surveillance except for Surveillance and Prevalence Studies; the subcategory of Model Systems/Therapeutic Targets under Treatments and Interventions, and; everything under Screening and Diagnosis except for the subcategory of Diagnostic and Screening Tools. These analyses revealed that 74% of 2013 research funding in the US was allocated to Basic Research, with Intervention comprising more than one-half of the funding allocated to Research on Practice and Policy.

While these analyses of trends in research publications and funding reveal a clear emphasis on basic research, they shed little light on meaningful changes over time. Does the arc of ASD research bend from understanding towards implementation, and eventually population-level impacts? The goal of the present study was to apply a research roadmap to publications in this journal during the 40 year period since the release of the DSM-III, to answer the following questions. Are there publication trends suggesting that basic research is being translated into important outcomes, as evidenced by increases in applied research relative to basic research, and in applied research evaluating specific interventions, especially those involving community-based programs?

## Methods

### Search Strategy

We used PSYCINFO to search all articles published in the Journal for Autism and Developmental Disorders (JADD) in 1979, 1989, 1999, 2009, and 2019, with search terms related to autism (aut*) in the title or abstract. The resulting dataset was scanned to exclude letters/commentaries, review articles, and meta-analyses. The remaining abstracts were reviewed to exclude case studies that relied primarily on clinical descriptions (e.g., Artemios et al., 2019; Wakabayashi, 1979) as opposed to measurable characteristics or outcomes. Assignment of the remaining articles to the categories outlined below was primarily conducted through a review of the abstract, with a full-text review as needed. Evidence for the involvement of community-based programs or professionals in intervention research studies was based on full-text review.

### Categories of Research

#### Applied versus Basic Research

Original research studies were considered to be applied if they sought to assess or achieve a specific clinical, behavioral, or educational outcome through services provided to a clinical population. This was most clearly evident when research helped to test a new assessment tool or intervention technique with people receiving services associated with their ASD. This also included other research described in greater detail below; for example, capturing other outcomes broadly associated with these services, barriers or other factors affecting access to these services, and so on. This excluded, however, research on a characteristic, and behavior without a clear and immediate clinical outcome. For example, research on a difference in the decoding of facial expressions was categorized as basic research unless there was clear evidence that this assessment has been successfully used to inform a clinical treatment plan in a clinical population.

In most cases, applied research focused on clinical populations. It excluded research on populations of individuals less likely to have sought or to be seeking help for their ASD. For example, studies that sought to validate new assessment procedures on typical or non-clinical populations (e.g., Jia et al., 2019) were categorized as basic research. Studies that screened for ASD in the broader population, to identify those eligible for early intervention, were included in applied research.

All other original empirical research studies were categorized as basic research. This included studies focused on the possible cause, characteristics (Aaron et al., 2019), correlates (Abbeduto et al., 2019), or course (Baghdadli et al., 2019) of ASD. Some studies that might have yielded findings interpreted as having important implications for assessment or intervention but that fell short of actually testing a specific procedure on a clinical population were also categorized under basic research.

#### Subcategories of Applied Research

Assessment research included all original studies that involved the application of a specific assessment procedure on a clinical population. This included all studies designed to establish the essential validity or reliability of an assessment procedure with a clinical population. Research meant to improve differentiation between different subgroups was considered here if it involved tools that a community-based practitioner might use. Studies that explored other characteristics of an assessment procedure without direct and immediate implications for practice were categorized as basic research (e.g., Adams et al., 2019). To be categorized under assessment research, the study had to involve the analysis of assessment data collected from data collected from and/or about a person with ASD. Studies that collected data on parents or service providers related to assessment services were categorized under other applied research.

Intervention Research included all studies that gathered and analyzed data collected on the changes in skills or behaviors observed in a clinical population of people with ASD following an intervention. Studies that collected data on parents or service providers related to intervention services were categorized under other applied research, unless this was part of a study in which they served as the interventionists.

Relevant sections of the Methods section were carefully reviewed for any indications that the study involved community-based programs (e.g., home, public school, and so on), suggesting a critical shift in the translation of outcome research into practice beyond traditional research settings and programs. Where the research was conducted (the setting) was distinguished from who delivered the intervention (the primary interventionist) because it is not uncommon for researchers to deliver the interventions in community settings, especially during a transition period when the efficacy of an intervention is not yet established. A third category was included, capturing intervention research studies conducted in hospital-based programs, whose status is ambiguous given that some hospitals partner with or house research centers themselves. If the study involved a range of participants, programs, or interventionists across different research and community settings, the study was categorized according to where the majority came from. In the absence of clear information, the program, setting, and interventionist were assumed to be research-based. The interventionist was categorized under research when researchers provided direct intervention or parent training, but not if their role was confined to initial training of other professionals, assessment of the participant, and/or fidelity checks.

Other Applied Research included any other research study that addressed other factors related to clinical assessment or intervention outcomes such as those assessing characteristics or outcomes of parents (Bourke-Taylor et al., 2019) or professionals, evaluating the quality of assessment or intervention, identifying clinical correlates of intervention outcomes, assessing satisfaction with intervention, and so on. This included research assessing knowledge about or attitudes towards specific assessment/intervention practices, but not about the overall climate of a clinical setting. This also included prevalence research of a specific diagnostic outcome if this was conducted at a population level.

## Results

**Fig. 1:**
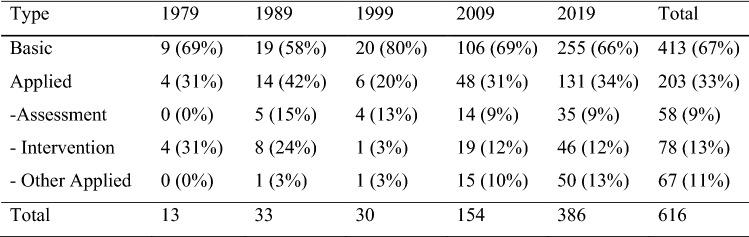
Number of original research studies published in JADD

The initial search yielded 708 citations in JADD across five time points (1979, 1989, 1999, 2009, 2019). A title/abstract review identified 92 citations that did not present original empirical research (e.g., letters/commentaries, review articles, meta-analyses, and so on). The classification of the remaining 616 citations on the research roadmap is summarized in Fig. 1.

Of the 413 basic and 204 applied research studies identified, more than 90% were published since 2009 and 60% in 2019 alone. There was no evidence of a relative increase in applied research; basic research remained steady throughout this period, ranging from between 58 and 69% except for a peak of 80% in 1999. Relative to other categories, intervention research dropped from a peak of 31% and 24% of all research in 1979 and 1989, to 12% in 2009 and 2019.

This decrease in intervention research in 2009 and 2019 corresponds with an increase in other applied research. After one applied research study in 1989 (Sugiyama & Abe, 1989) and a second in 1999 (Kasari et al., 1999), other applied research studies outstripped assessment research by 2009 and intervention research by 2019. This category included a range of studies, examining shifts in diagnostic practices (Grether et al., 2009), surveys of treatment practices (Wong, 2009), the use of web-based tools and supports (Mazurek et al., 2019), and parent perspectives on assessment (Jashar et al., 2019) and intervention (Grindle et al., 2009), among other themes.

**Fig. 2:**
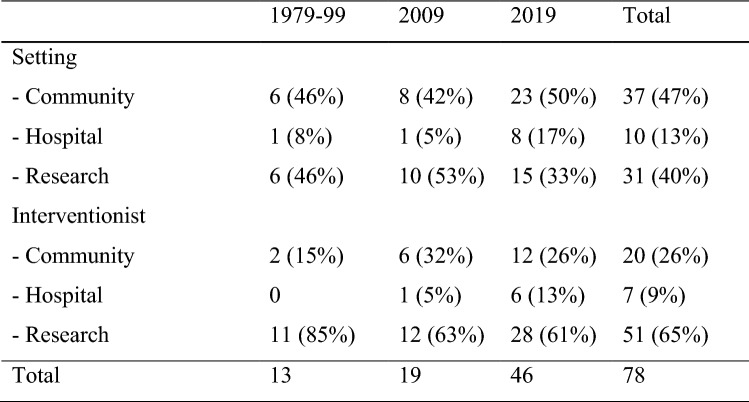
Intervention research conducted in community-based programs

With regards to the translation of intervention research into community-based programs data from the first three time periods were collapsed together because of the low number of outcome research studies recorded. These results are summarized in Fig. 2. The proportion of intervention research studies occurring in community settings remained steady throughout this period, between 42 and 50%. While the proportion of intervention research studies occurring in more traditional research settings decreased from 53 to 33% between 2009 and 2019, this was partially due to an increase in hospital-based intervention research. Given the many hospital-based programs that are either research centers or research partners themselves, it is difficult to determine whether this reflects a true shift away from traditional research settings. Overall, only about 6% of all original research studies published in JADD clearly involved intervention research conducted in community settings, a proportion that did not increase during the study period.

Almost two-thirds of the direct intervention was carried out by researchers or their students or staff. This proportion decreased from a high of 85% during the first three time periods to 61% by 2019. This split reflects that interventions in a community setting (e.g., in the home or school) may have not only required that researchers play a critical role in training and supervising the delivery of an intervention, but sometimes also in delivering it themselves. Overall, only about 3–4% of all original research studies published in JADD clearly involved research in which community-based practitioners clearly delivered the intervention. There was no notable decrease over the course of the study period in the overall proportion of published studies in which researchers delivered the intervention.

## Discussion

In this study of research published since the release of the DSM-III in 1980, we sought evidence of a relative increase in the kinds of applied research more likely to immediately impact the lives of people living with ASD. After categorizing more than 600 original empirical research studies published in this journal at five different points as either involving basic or applied research, there was no clear evidence of a shift in the proportion of applied research over time. The proportion of basic research decreased following a peak of 80% in 1999, but only to points higher than had been noted at the two earliest time points in this study. While the absolute number of applied research studies also increased dramatically over these most recent time points, the number of basic research studies did so too, with basic research continuing to make up about two-thirds of all publications. The recent explosion in research publications has probably increased our general knowledge about ASD – its possible causes, characteristics, and general trajectory – but this does not appear to have spurred a relatively increased interest in the more practical knowledge about how to help people living with ASD. In other words, the arc of ASD research does not appear to have bent towards impact over the past 40 years.

To understand this explosion of publications, it is worth considering the relationship between the publication and the funding of empirical research related to ASD. This emphasis on basic research in publication patterns is certainly consistent with funding patterns described earlier in the UK (Pellicano et al., 2013) and re-analyses of research funding in the US between 2008 and 2013 (Doehring, 2019b). This explosion in research publications corresponds with passage of the Combating Autism Act in 2008 (and continued through subsequent re-authorizations), as well as the increased efforts of organizations like Autism Speaks and the Simons Foundation to raise and direct hundreds of millions of dollars from private sources.

It is also important to recognize that relationships between research funding and publication patterns may exist at multiple levels. At the system level, research funding and research publications are probably closely interwoven. While these dramatic increases in publications in 2009 and 2019 are certainly the fruits of these investments in research, it also seems likely that research published in the decade prior to this period fueled the advocacy that led to the Combating Autism Act. For example, earlier research published by the Centers for Disease Control and suggesting higher than expected prevalence (Bertrand et al., 2001) led to increased calls for more research to understand and respond to these trends. For the individual researcher, research publications and research funding are inextricably interwoven. Researchers early in their career without a strong record of research publications will struggle to secure the research funding needed to secure an academic appointment, while those unable to translate research funding into still more publications may find it difficult to build a program of study. To some extent, this explosion in research publications since 2009 might therefore be attributed to a new generation of researchers eager to quickly establish their careers, encouraged by a new influx of funding.

In this context, the relatively decreased emphasis on intervention research is surprising. Over the 40-year period studied here, the emphasis has shifted away from intervention research and towards basic research. By 2009, only one out of every eight studies tested an intervention, a decrease of 50% relative to 20 years earlier. The design of the current study sheds little light on the reasons for this disconnection. One possibility is that some types of basic research have not spawned specific interventions, only more basic research. For example, while some the evidence-based interventions identified through the systematic reviews of Odom and his colleagues (Wong et al., 2015) can trace their origins to basic research on the social, communicative and behavioral characteristics of children with ASD, it is not clear whether any of these intervention practices can be traced back to other basic research findings related to neurological or genetic differences. It is also possible that interventions resulting from basic research have not led to other intervention research studies, but this seems less likely. Systematic reviews of evidence-based practices (Reichow et al., 2011) reveal the many different types of research studies that may follow an initial publication of an effective intervention; other research studies can replicate the original finding, further specify the skill or behavior targeted, explore outcomes in other populations, test training protocols, and so on. In many respects, the initial publication of an effective intervention should trigger a new wave of related research, and result in a relative increase in related publications.

It is also possible that these differences simply reflect that intervention research can be more difficult to do. Stated simply, basic research requires identifying and then evaluating an interesting characteristic in an interesting population under very carefully controlled circumstances. Interventions add other layers of complexity to control for; the need to identify a clinical need, access a clinical population, deliver an intervention with fidelity, and so on. Both the design and the implementation of intervention research require that the researcher has either invested the time needed to become an expert in the intervention, or has identified a clinical partner with the requisite expertise. Extremely rigorous standards of research design (e.g., including double-blind, placebo controlled clinical trials) create additional barriers. It is therefore easy to imagine that researchers faced with these barriers will either publish fewer articles describing intervention research, or avoid intervention research altogether. Early career researchers under pressure to publish may hesitate to invest the hours needed to build broad clinical expertise.

Given the relative lack of focus on intervention research, the lack of a clear shift towards intervention research outside of traditional research settings is hardly surprising. Overall, only about 3% of original empirical research that was published in JADD tested interventions in community programs. About one-half of intervention research studies clearly occurred in community settings, and only about only half of these clearly relied on community-based interventionists. These proportions did not consistently increase over the time periods studied. The increases in research conducted hospital-based settings may, however, merit closer examination. While there are many examples of hospital-based professionals and programs so closely intertwined with research centers that they ae best considered a traditional research setting, the number of studies produced by hospital-based professionals who devote most of their activities to clinical care and who conduct research largely independent of research centers is less clear.

What are other possible reasons for this gap in community-based research? Conducting intervention research in community settings adds still more layers of complexity for investigators to consider. Partner agencies in the community must be identified that see the value of research and the intervention under consideration. Interventionists must become partners in delivering the intervention with fidelity. Engaging programs and training interventionists requires additional time and resources, creating more pressure for researchers. If it is easy to imagine why a researcher might turn away from pursuing intervention research, it is even easier to imagine why they would hesitate to conduct intervention research outside of a traditional research setting, especially given the upfront investments in the time needed to build relationships with community-based programs. Given the pressures to publish, an aspiring clinical researcher might hesitate to seek the kind of practical experience delivering community-based interventions needed to anticipate the challenges of conducting research in these settings.

The rise of other applied research over the most recent time periods is interesting, and worthy of additional study. Some of the studies sought to understand other gaps in early identification in the community – like the difference between clinically- and research identified cohorts (Barbaresi et al., 2009), referral biases amongst ethnic minorities (Begeer et al., 2009), or shifts in autism identification within state agencies (Grether et al., 2009). These represent a natural extension of efforts to improve identification in research setting. Otherwise, the range of topics considered was very broad, and not as easily understood.

Given this special issue on the 40th anniversary of the DSM-III, the pattern of research surrounding the release of the DSM-IV in 1994 is particularly interesting. The fact that assessment research peaked 5 years prior to and 5 years after the release of the DSM-IV may reflect how this research helped to first drive the development of a new taxonomy and then sought to understand its implications. Seminal publications introducing the Autism Diagnostic Observation Schedule (ADOS) (Lord et al., 1989) and the Autism Diagnostic Interview (ADI) (Le Couteur et al., 1989), 1989 marked a watershed in diagnosis. Basic research peaked 5 years after the release of the DSM-IV, exploring a new taxonomy and the spectrum of ASD with designs that distinguished between subtypes in relation to associated features (Craig & Baron-Cohen, 1999; Dennis et al., 1999; Klin et al., 1999; Müller et al., 1999). As a result, interest shifted away from intervention, with only one such study published in 1999 (Bolman & Richmond, 1999).

One way to determine if and how the arc of science bends towards impact is to consider patterns of publication across an entire field, as described above. It is also interesting to consider if and how specific findings from these early years have since shaped practice. For example, the emergence of the ADOS and ADI in 1989 clearly set new standards for diagnosis in research settings. No other instruments have arguably played a greater role in shaping subsequent iterations of the DSM. Their record in shaping the identification of ASD in community settings is, however, less certain. While some of the larger and more specialized community programs can justify the investments needed to build clinical expertise in the use of these instruments (Doehring & Winterling, 2011), especially given the volume and complexity of cases encountered, these instruments have not had nearly the same breadth of impact on diagnosis in most community settings. Indeed, the definitions of ASD included in many state and federal regulations that guide the identification of ASD in public schools remain woefully outdated (Doehring & Volkmar, 2016).

It is also interesting to consider the four intervention studies published in 1979. Three of these (Holman & Baer, 1979; Koegel et al., 1979; Strain et al., 1979) described specific behavioral procedures to improve the acquisition of important social and academic skills while decreasing problem behavior, presaging trends that would continue for decades. These kinds of behaviorally-based teaching strategies have since transformed home-and school-based programs across the globe. When informed by the functional assessment of problematic behavior, these techniques can also reduce the occurrence of behaviors that otherwise would have prevented countless children with ASD from accessing community-based settings.

In contrast, the fourth intervention study published in 1979 included punishment in a package of techniques intended to suppress behaviors that are highly problematic, in two institutionalized adults with very significant levels of disability (Foxx et al., 1979). On the one hand, the shift away from punishment towards positive and preventative interventions informed by the function of behavior, as captured by studies included in this review (Carr & Kemp, 1989; Duker & Rasing, 1989), demonstrates the potential for research to have tremendous impacts. On the other hand, reports in the popular press continue to document the inappropriate use of seclusion and restraint to effectively punish problematic behaviors, sometimes with deadly consequences. Nonetheless, interventions to address these types of severe behaviors are rarely studied. One systematic review of all research published over an 18 year period on interventions targeting severe behaviors identified only about 100 studies involving a total of about 150 participants (Doehring et al., 2013). More recent reviews continue to highlight the lack of interest in individuals with the most significant levels of disability (Stedman et al., 2018). Adults like those studied by Foxx and his colleagues more than 40 years ago live with the most profound consequences of their disability, and have the most to benefit from improved understanding and treatment. In these cases, the arc of research appears to bend away from those with the most to gain.

The systematic mapping of research in a specific area of assessment or intervention might provide other insights into how basic research can be translated into population-level impacts. Consider the preliminary application of a research roadmap to the CHAT and its variants (﻿Doehring, 2019a). Basic research was first used to describe specific deficits in joint attention and other social-communicative behaviors among young children already identified with ASD (Sigman et al., 1986). Research subsequently tested whether these and other deficits could become the basis for an assessment tool, like a checklist to identify ASD amongst children at high risk but yet to be diagnosed (Baron-Cohen et al., 1992). Research then helped to confirm that such a checklist could be successfully utilized at a larger scale, outside of a traditional research setting, by community-based practitioners (Baron-Cohen et al., 1996). Next, research sought to modify this checklist to further improve its validity, reliability, and ease of use (Robins et al., 2001). Later research demonstrated that this modified checklist could be successfully translated for use across a range of languages and cultures (Albores-Gallo et al., 2012; Canal-Bedia et al., 2011; Inada et al., 2011) and incorporated into assessment protocols commonly used by community-based practitioners (Glascoe et al., 2007). Three decades after specifically relevant basic research had been first published, new studies have begun to show how such a checklist might increase access to early intervention when combined with other policy changes (Rotholz et al., 2017).

Research roadmaps like those modelled here should be situated within larger roadmaps that integrate research with services, training, policy, and advocacy, and that translate all of this into action at the local regional, state, and national level (Doehring, 2013). We have applied these roadmaps to a fictional case study of a young man in behavioral crisis, to illustrate how breakdowns in the services and supports available in local, regional, and state agencies can lead to a potentially lifelong but completely preventable placement in an institutional setting (Doehring, 2014). We have also explored the interface between research and policy at the national level (Doehring, 2019b), and found no examples of research undertaken in an intentional, coordinated manner to influence national or even state policy.

Several important limitations of the present analyses are worth noting here. For example, this review focused on publications in JADD. This was necessary given that JADD was the only journal focused on ASD during the time period studied, since the publication of the DSM-III. There are other journals that have since emerged, however, that also focus on ASD, that include research on other populations that sometimes overlap with ASD (e.g., people with I/DD), and that have published research involving people with ASD from the perspective of specific disciplines (Behavior Analysis, Pediatrics, Education, Public Health, and so on). It will be important for future research to confirm whether these same patterns hold true regardless of the journal in which this research was published.

It is legitimate to question whether the percentage of publications adequately captures the arc of science. Two publications might vary greatly with respect to the number of participants, and the significance of the results. Changing publication standards could confound comparisons across different time points, although many of the patterns noted here remained relatively consistent over the decades. The number of citations for a given class of studies might offer a different metric, capturing what researchers are interested in. To the extent that research depends heavily on funding, and that funding is generally directed towards the proposals determined to be most likely to advance understanding, analyses of patterns of funding across different types of research should reflect what researchers and policymakers believe is most important and are ready to invest in. We hope that the present study will spur a debate about each of these potential metrics for the impact of science.

These roadmaps, together with the findings of the present review, raise important questions about research publications, research funding, and the training of researchers that might be answered through future study. How often is basic research conducted with a specific goal to improve assessment or intervention? To what extent are these efforts driven by researchers with broad clinical training and/or experience? How often does such research actually result in a testable clinical practice? How often do researchers seeking to improve community-based outcomes have experience delivering services in community-based programs? Such gaps in the types of training and experience of researchers can have impacts beyond the specific research itself, by shaping the culture and the structure of the research enterprise, and perhaps contributing to the gaps in intervention research noted above. Without such experience, for example, those reviewing intervention research studies for publication might inadvertently set unreasonable expectations, because they do not recognize challenges inherent to such research, especially when it is conducted in community settings. Likewise, those reviewing proposals for funding might penalize the clinical researcher whose publication record might be less impressive because of the time invested broadening their clinical expertise outside of a traditional research setting. Assigning greater importance to intervention research publications and real-world clinical experience and outcomes may increase incentives for the next generation of researchers to design programs of study to bend the arc of science back towards impact.

## References

[CR1] Aaron E, Montgomery A, Ren X, Guter S, Anderson G, Carneiro AMD, Veenstra-VanderWeele J (2019). Whole blood serotonin levels and platelet 5-HT(2A) binding in autism spectrum disorder. Journal of Autism and Developmental Disorders.

[CR2] Abbeduto L, Thurman AJ, McDuffie A, Klusek J, Feigles RT, Ted Brown W, Roberts JE (2019). ASD comorbidity in fragile X syndrome: Symptom profile and predictors of symptom severity in adolescent and young adult males. Journal of Autism and Developmental Disorders.

[CR3] Adams D, Paynter J, Clark M, Roberts J, Keen D (2019). The developmental behaviour checklist (DBC) profile in young children on the autism spectrum: The impact of child and family factors. Journal of Autism and Developmental Disorders.

[CR4] Albores-Gallo L, Roldan-Ceballos O, Villarreal-Valdes G, Betanzos-Cruz BX, Santos-Sanchez C, Martinez-Jaime MM, Hilton CL (2012). M-CHAT Mexican version validity and reliability and some cultural considerations. ISRN Neurology.

[CR5] Artemios P, Areti S, Katerina P, Helen F, Eirini T, Charalambos P (2019). Autism spectrum disorder and psychiatric comorbidity in a patient with myhre syndrome. Journal of Autism and Developmental Disorders.

[CR6] Baghdadli A, Rattaz C, Michelon C, Pernon E, Munir K (2019). Fifteen-year prospective follow-up study of adult outcomes of autism spectrum disorders among children attending centers in five regional departments in France: The EpiTED cohort. Journal of Autism and Developmental Disorders.

[CR7] Bailey AJ (2009). Where are the autism economists?. Autism Research.

[CR8] Barbaresi WJ, Colligan RC, Weaver AL, Katusic SK (2009). The incidence of clinically diagnosed versus research-identified autism in Olmsted County, Minnesota, 1976–1997: Results from a retrospective, population-based study. Journal of Autism and Developmental Disorders.

[CR9] Baron-Cohen S, Allen J, Gillberg C (1992). Can autism be detected at 18 months? The needle, the haystack, and the CHAT. The British Journal of Psychiatry.

[CR10] Baron-Cohen S, Cox A, Baird G, Swettenham J, Nightingale N, Morgan K, Charman T (1996). Psychological markers in the detection of autism in infancy in a large population. The British Journal of Psychiatry.

[CR11] Begeer S, Bouk SE, Boussaid W, Terwogt MM, Koot HM (2009). Underdiagnosis and referral bias of autism in ethnic minorities. Journal of Autism and Developmental Disorders.

[CR12] Bertrand J, Mars A, Boyle C, Bove F, Yeargin-Allsopp M, Decoufle P (2001). Prevalence of autism in a United States population: The brick township, New Jersey, investigation. Pediatrics.

[CR13] Bettelheim B (1967). The empty fortress: Infantile autism and the birth of the self.

[CR14] Bolman WM, Richmond JA (1999). A double-blind, placebo-controlled, crossover pilot trial of low dose dimethylglycine in patients with autistic disorder. Journal of Autism and Developmental Disorders.

[CR15] Bourke-Taylor HM, Jane F, Peat J (2019). Healthy mothers healthy families workshop intervention: A preliminary investigation of healthy lifestyle changes for mothers of a child with a disability. Journal of Autism and Developmental Disorders.

[CR16] Canal-Bedia R, Garcia-Primo P, Martin-Cilleros MV, Santos-Borbujo J, Guisuraga-Fernandez Z, Herraez-Garcia L, Posada-de la Paz M (2011). Modified checklist for autism in toddlers: Cross-cultural adaptation and validation in Spain. Journal of Autism and Developmental Disorders.

[CR17] Carr EG, Kemp DC (1989). Functional equivalence of autistic leading and communicative pointing: Analysis and treatment. Journal of Autism and Developmental Disorders.

[CR18] Craig J, Baron-Cohen S (1999). Creativity and imagination in autism and Asperger syndrome. Journal of Autism and Developmental Disorders.

[CR19] Dennis M, Lockyer L, Lazenby AL, Donnelly RE, Wilkinson M, Schoonheyt W (1999). Intelligence patterns among children with high-functioning autism, phenylketonuria, and childhood head injury. Journal of Autism and Developmental Disorders.

[CR56] Doehring P (2013). Autism services across america: Road maps for improving state and national education, research, and training programs.

[CR57] Doehring P, Volkmar F, Paul R, Rogers S, Pelphrey K (2014). Translating research into effective social policy. Handbook of autism and pervasive developmental disorders: Volume 2, assessment, interventions, and policy.

[CR59] Doehring, P. (2019a). A systematic review of ASD screening from basic research to population impact: A model research roadmap? International Society for Autism Research (INSAR), 2019 annual meeting, Poster 30172, Montreal, Canada, May 2019.

[CR60] Doehring P, Volkmar FR (2019). The impact of ASD research on national policy: Lessons from the combating autism act and the national institute for health and care excellence. Autism and pervasive developmental disorders.

[CR20] Doehring P, Reichow B, Palka T, Phillips C, Hagopian L (2013). Behavioral approaches to managing severe problem behaviors in children with autism-spectrum and related developmental disorders: A descriptive analysis. Child and Adolescent Psychiatric Clinics of North America.

[CR58] Doehring P, Volkmar FR (2016). Knowledge gaps in ASD research: Short and long term implications for policy. Journal of Autism and Developmental Disorders.

[CR21] Doehring P, Winterling V, Reichow B, Doehring P, Cicchetti DV, Volkmar FR (2011). The implementation of evidence-based practices in public schools. Evidence-based practices and treatments for children with autism.

[CR22] Duker PC, Rasing E (1989). Effects of redesigning the physical environment on self-stimulation and on-task behavior in three autistic-type developmentally disabled individuals. Journal of Autism and Developmental Disorders.

[CR23] Foxx RM, Snyder MS, Schroeder F (1979). A food satiation and oral hygiene punishment program to suppress chronic rumination by retarded persons. Journal of Autism and Developmental Disorders.

[CR24] Glascoe FP, Macias MM, Wegner LM, Robertshaw NS (2007). Can a broadband developmental-behavioral screening test identify children likely to have autism spectrum disorder?. Clinical Pediatrics.

[CR25] Grether JK, Rosen NJ, Smith KS, Croen LA (2009). Investigation of shifts in autism reporting in the California department of developmental services. Journal of Autism and Developmental Disorders.

[CR26] Grindle CF, Kovshoff H, Hastings RP, Remington B (2009). Parents' experiences of home-based applied behavior analysis programs for young children with autism. Journal of Autism and Developmental Disorders.

[CR27] Holman J, Baer DM (1979). Facilitating generalization of on-task behavior through self-monitoring of academic tasks. Journal of Autism and Developmental Disorders.

[CR28] Inada N, Koyama T, Inokuchi E, Kuroda M, Kamio Y (2011). Reliability and validity of the Japanese version of the modified checklist for autism in toddlers (M-CHAT). Research in Autism Spectrum Disorders.

[CR29] Interagency Autism Coordinating Committee (IACC). (2020a). IACC strategic plan for autism spectrum disorder (ASD) 2018–19 update. July 2020*.* Retrieved from the U.S. Department of Health and Human Services Interagency Autism Coordinating Committee website http://iacc.hhs.gov/strategic-plan/2019/

[CR30] Interagency Autism Coordinating Committee (IACC). (2020b). 2019 IACC summary of advances in autism spectrum disorder research. May 2020. Retrieved from the U.S. Department of Health and Human Services Interagency Autism Coordinating Committee website https://iacc.hhs.gov/publications/summary-of-advances/2019/

[CR31] Jashar DT, Fein D, Berry LN, Burke JD, Miller LE, Barton ML, Dumont-Mathieu T (2019). Parental perceptions of a comprehensive diagnostic evaluation for toddlers at risk for autism spectrum disorder. Journal of Autism and Developmental Disorders.

[CR32] Jia R, Steelman ZR, Jia HH (2019). Psychometric assessments of three self-report autism scales (AQ, RBQ-2A, and SQ) for general adult populations. Journal of Autism and Developmental Disorders.

[CR33] Kasari C, Freeman SF, Bauminger N, Alkin MC (1999). Parental perspectives on inclusion: Effects of autism and down syndrome. Journal of Autism and Developmental Disorders.

[CR34] Klin A, Sparrow SS, de Bildt A, Cicchetti DV, Cohen DJ, Volkmar FR (1999). A normed study of face recognition in autism and related disorders. Journal of Autism and Developmental Disorders.

[CR35] Koegel RL, Schreibman L, Britten K, Laitinen R (1979). The effects of schedule of reinforcement on stimulus overselectivity in autistic children. Journal of Autism and Developmental Disorders.

[CR36] Le Couteur A, Rutter M, Lord C, Rios P, Robertson S, Holdgrafer M, McLennan J (1989). Autism diagnostic interview: A standardized investigator-based instrument. Journal of Autism and Developmental Disorders.

[CR37] Lord C, Rutter M, Goode S, Heemsbergen J, Jordan H, Mawhood L, Schopler E (1989). Autism diagnostic observation schedule: A standardized observation of communicative and social behavior. Journal of Autism and Developmental Disorders.

[CR38] Mazurek MO, Curran A, Burnette C, Sohl K (2019). ECHO autism STAT: Accelerating early access to autism diagnosis. Journal of Autism and Developmental Disorders.

[CR39] Müller RA, Behen ME, Rothermel RD, Chugani DC, Muzik O, Mangner TJ, Chugani HT (1999). Brain mapping of language and auditory perception in high-functioning autistic adults: A PET study. Journal of Autism and Developmental Disorders.

[CR40] Office of Autism Research Coordination (OARC), National Institute of Mental Health and Thomson Reuters, Inc. on behalf of the Interagency Autism Coordinating Committee (IACC). (2012). IACC/OARC autism spectrum disorder research publications analysis report: The global landscape of autism research. July 2012. Retrieved from the Department of Health and Human Services Interagency Autism Coordinating Committee website http://iacc.hhs.gov/publications-analysis/july2012/index.shtml

[CR41] Office of Autism Research Coordination, National Institute of Mental Health, on behalf of the Interagency Autism Coordinating Committee (IACC). (2019). 2016 IACC autism spectrum disorder research portfolio analysis report. January 2019. Retrieved from the U.S. Department of Health and Human Services Interagency Autism Coordinating Committee website https://iacc.hhs.gov/portfolio-analysis/2016/index.shtml

[CR42] Office of Autism Research Coordination, National Institute of Mental Health, on behalf of the Interagency Autism Coordinating Committee (IACC). (2017). 2013 IACC autism spectrum disorder research portfolio analysis report. April 2017. Retrieved from the U.S. Department of Health and Human Services Interagency Autism Coordinating Committee website https://iacc.hhs.gov/portfolio-analysis/2013/index.shtml

[CR43] Pellicano E, Dinsmore A, Charman T (2013). A future made together: Shaping autism research in the UK.

[CR44] Reichow B, Doehring P, Cicchetti DV, Volkmar FR (2011). Evidence-based practices and treatments for children with autism.

[CR45] Robins DL, Fein D, Barton ML, Green JA (2001). The modified checklist for autism in toddlers: An initial study investigating the early detection of autism and pervasive developmental disorders. Journal of Autism and Developmental Disorders.

[CR46] Rotholz DA, Kinsman AM, Lacy KK, Charles J (2017). Improving early identification and intervention for children at risk for autism spectrum disorder. Pediatrics.

[CR47] Sigman MD, Mundy P, Sherman T, Ungerer J (1986). Social interactions of autistic, mentally retarded and normal children and their caregivers. Journal of Child Psychology and Psychiatry and Allied Disciplines.

[CR48] Stedman, A., Taylor, B., Erard, M., Peura, C., & Siegel, M. (2018). Are children severely affected by autism spectrum disorder underrepresented in treatment studies? An analysis of the literature. Journal of Autism and Developmental Disorders. 10.1007/s10803-018-3844-y 10.1007/s10803-018-3844-y PMC6450830 30536112

[CR49] Strain PS, Kerr MM, Ragland EU (1979). Effects of peer-mediated social initiations and prompting/reinforcement procedures on the social behavior of autistic children. Journal of Autism and Developmental Disorders.

[CR50] Sugiyama T, Abe T (1989). The prevalence of autism in Nagoya, Japan: A total population study. Journal of Autism and Developmental Disorders.

[CR51] U.S. Department of Health and Human Services, National Institutes of Health, Office of Autism Research Coordination (On behalf of the Office of the Secretary) (2019). Report to congress on activities related to autism spectrum disorder and other developmental disabilities, under the autism collaboration, accountability, research, education and support (Autism CARES) Act of 2014. March 2019. Retrieved from the U.S. Department of Health and Human Services Interagency Autism Coordinating Committee website https://iacc.hhs.gov/publications/report-to-congress/2018/

[CR52] Wakabayashi S (1979). A case of infantile autism associated with down’s syndrome. Journal of Autism and Developmental Disorders.

[CR53] Wong C, Odom SL, Hume KA, Cox AW, Fettig A, Kucharczyk S, Schultz TR (2015). Evidence-based practices for children, youth, and young adults with autism spectrum disorder: A comprehensive review. Journal of Autism and Developmental Disorders.

[CR54] Wong VC (2009). Use of complementary and alternative medicine (CAM) in autism spectrum disorder (ASD): Comparison of Chinese and western culture (Part A). Journal of Autism and Developmental Disorders.

[CR55] Zwicker JD, Emery JCH (2014). Autism research funding allocation: Can economics tell us if we have got it right?. Autism Research.

